# Decline in SARS-CoV-2 Antibodies After Mild Infection Among Frontline Health Care Personnel in a Multistate Hospital Network — 12 States, April–August 2020

**DOI:** 10.15585/mmwr.mm6947a2

**Published:** 2020-11-27

**Authors:** Wesley H. Self, Mark W. Tenforde, William B. Stubblefield, Leora R. Feldstein, Jay S. Steingrub, Nathan I. Shapiro, Adit A. Ginde, Matthew E. Prekker, Samuel M. Brown, Ithan D. Peltan, Michelle N. Gong, Michael S. Aboodi, Akram Khan, Matthew C. Exline, D. Clark Files, Kevin W. Gibbs, Christopher J. Lindsell, Todd W. Rice, Ian D. Jones, Natasha Halasa, H. Keipp Talbot, Carlos G. Grijalva, Jonathan D. Casey, David N. Hager, Nida Qadir, Daniel J. Henning, Melissa M. Coughlin, Jarad Schiffer, Vera Semenova, Han Li, Natalie J. Thornburg, Manish M. Patel, CDC COVID-19 Response Team, IVY Network, CDC COVID-19 Response Team, Mohammed Ata Ur Rasheed, Lisa Mills, Sandra N. Lester, Brandi Freeman, Bailey Alston, Muyiwa Ategbole, Peter Browning, Shanna Bolcen, Darbi Boulay, Li Cronin, Ebenezer David, Rita Desai, Monica Epperson, Yamini Gorantla, Tao Jia, Pete Maniatis, Kristina Ortiz, So Hee Park, Palak Patel, Yunlong Qin, Heather Tatum, Briana Zellner, IVY Network, Adrienne Baughman, Kimberly W. Hart, Robert McClellan, Rendie McHenry, Jakea Johnson, Andrea Fletcher, Kemberlyne Cordero, Lori Kozikowski, Lesley De Souza, Sarah Romain, Scott Ouellette, Andres Santana, Sherell Thornton-Thompson, Michelle Howell, Jennifer Peers, Shelby Shelton, Lani Finck, Kirsten Soules, Michael Klausner, Ximena Calderon-Morales, Heidi L. Erickson, Audrey Hendrickson, Jamie Stang, Ellen Maruggi, Alex Dunn, Eddie Stenehjem, Valerie Aston, Mikaele Bown, Michelle Matheu, Rilee Smith, Olivia Krol, Andrew Salar, Makrina Kamel, Kelly Nguyen, Peter Huynh, Sarah Karow, Michelle Bright, Holly Bookless, Sandy Mullins, Kelly Neidert, Dina McGowan, Elizabeth Cassandra, Emily Brown, Claire Carlin, Trina Wemlinger, Breona Edwards, Lori Flores, Mary LaRose, Kathie J. Ferbas, Rachel Martin-Blais, Grace M. Aldrovandi, Olivia Thompson, Sakshi Sehgal

**Affiliations:** ^1^Vanderbilt University Medical Center, Nashville, Tennessee; ^2^CDC COVID-19 Response Team; ^3^Baystate Medical Center, Springfield, Massachusetts; ^4^Beth Israel Deaconess Medical Center, Boston, Massachusetts; ^5^University of Colorado School of Medicine, Aurora, Colorado; ^6^Hennepin County Medical Center, Minneapolis, Minnesota; ^7^Intermountain Medical Center and University of Utah School of Medicine, Salt Lake City, Utah; ^8^Montefiore Medical Center, Bronx, New York; ^9^Oregon Health & Science University Hospital, Portland, Oregon; ^10^Ohio State University Wexner Medical Center, Columbus, Ohio; ^11^Wake Forest University Baptist Medical Center, Winston-Salem, North Carolina; ^12^Johns Hopkins Hospital, Baltimore, Maryland; ^13^UCLA Medical Center, Los Angeles, California; ^14^Harborview Medical Center, Seattle, Washington.; CDC COVID-19 Response Team; CDC COVID-19 Response Team; CDC COVID-19 Response Team; CDC COVID-19 Response Team; CDC COVID-19 Response Team; CDC COVID-19 Response Team; CDC COVID-19 Response Team; CDC COVID-19 Response Team; CDC COVID-19 Response Team; CDC COVID-19 Response Team; CDC COVID-19 Response Team; CDC COVID-19 Response Team; CDC COVID-19 Response Team; CDC COVID-19 Response Team; CDC COVID-19 Response Team; CDC COVID-19 Response Team; CDC COVID-19 Response Team; CDC COVID-19 Response Team; CDC COVID-19 Response Team; CDC COVID-19 Response Team; CDC COVID-19 Response Team; CDC COVID-19 Response Team.; IVY Network; IVY Network; IVY Network; IVY Network; IVY Network; IVY Network; IVY Network; IVY Network; IVY Network; IVY Network; IVY Network; IVY Network; IVY Network; IVY Network; IVY Network; IVY Network; IVY Network; IVY Network; IVY Network; IVY Network; IVY Network; IVY Network; IVY Network; IVY Network; IVY Network; IVY Network; IVY Network; IVY Network; IVY Network; IVY Network; IVY Network; IVY Network; IVY Network; IVY Network; IVY Network; IVY Network; IVY Network; IVY Network; IVY Network; IVY Network; IVY Network; IVY Network; IVY Network; IVY Network; IVY Network; IVY Network; IVY Network; IVY Network; IVY Network; IVY Network; IVY Network; IVY Network; IVY Network.

Most persons infected with SARS-CoV-2, the virus that causes coronavirus disease 2019 (COVID-19), develop virus-specific antibodies within several weeks, but antibody titers might decline over time. Understanding the timeline of antibody decline is important for interpreting SARS-CoV-2 serology results. Serum specimens were collected from a convenience sample of frontline health care personnel at 13 hospitals and tested for antibodies to SARS-CoV-2 during April 3–June 19, 2020, and again approximately 60 days later to assess this timeline. The percentage of participants who experienced seroreversion, defined as an antibody signal-to-threshold ratio >1.0 at baseline and <1.0 at the follow-up visit, was assessed. Overall, 194 (6.0%) of 3,248 participants had detectable antibodies to SARS-CoV-2 at baseline (*1*). Upon repeat testing approximately 60 days later (range = 50–91 days), 146 (93.6%) of 156 participants experienced a decline in antibody response indicated by a lower signal-to-threshold ratio at the follow-up visit, compared with the baseline visit, and 44 (28.2%) experienced seroreversion. Participants with higher initial antibody responses were more likely to have antibodies detected at the follow-up test than were those who had a lower initial antibody response. Whether decay in these antibodies increases risk for reinfection and disease remains unanswered. However, these results suggest that serology testing at a single time point is likely to underestimate the number of persons with previous SARS-CoV-2 infection, and a negative serologic test result might not reliably exclude prior infection.

Once infected with SARS-CoV-2, most persons develop virus-specific antibodies within 2–3 weeks (*2*,*3*). Serology tests are now being used widely in seroprevalence studies to understand patterns of viral spread, cumulative incidence of SARS-CoV-2 infection, and pandemic trajectory (*4*–*6*). Further, serologic testing has been proposed as a way to identify persons who might have developed immunity through a previous infection. Understanding how rapidly SARS-CoV-2 antibody levels decline after seroconversion is critical for interpreting serology results. A limited number of studies have found declines in SARS-CoV-2 antibody levels over time (*7*–*9*), but the frequency and timing of seroreversion (the decline in antibody levels below the positivity threshold after initial seroconversion) remains largely unknown.

The Influenza Vaccine Effectiveness in the Critically Ill (IVY) Network, a collaboration of academic medical centers in the United States that studies influenza and COVID-19 (*1*), enrolled a convenience sample of frontline health care personnel at 13 centers in 12 states,^†^ with a target of 250 participants per center. Health care personnel were eligible if they reported regular direct contact with COVID-19 patients and worked in the emergency department, intensive care unit, or other hospital-based unit that cared for patients with COVID-19. Participants underwent two study visits: a baseline visit (conducted April 3–June 19, 2020) and a follow-up visit approximately 60 days after the baseline visit. At both visits, blood was collected for SARS-CoV-2 antibody testing, and participants were questioned about demographic characteristics, underlying medical conditions, signs or symptoms of an acute viral infection from February 1, 2020, until the visit date,^§^ and any previous SARS-CoV-2 testing (e.g., reverse transcription–polymerase chain reaction [RT-PCR]) for acute infection. Blood specimens collected at the baseline and follow-up visits were tested for SARS-CoV-2 antibodies at CDC using an enzyme-linked immunosorbent assay (ELISA) against the extracellular domain of the SARS-CoV-2 spike protein (*4*). The assay detects all SARS-CoV-2 immunoglobulin (Ig) types (IgA, IgM, or IgG). Specimens were considered reactive with a signal-to-threshold ratio >1.0 at a background corrected serum dilution of 1:100, with higher ratios indicating higher antibody titers. The assay has a sensitivity estimated at 96% and specificity at 99% (*4*).

The change in signal-to-threshold ratio between the baseline visit and follow-up visit was quantified, and the percentage of participants who experienced seroreversion was reported. Logistic regression was used to evaluate the association between baseline signal-to-threshold value and seroreversion, adjusting for age, sex, race/ethnicity, number of days between the baseline and follow-up visit, and presence of one or more chronic medical condition. Analyses were conducted using Stata (version 16; StataCorp). The project was determined to be nonresearch public health surveillance by participating institutions and CDC and was conducted consistent with applicable federal law and CDC policy.^¶^

Among 3,248 health care personnel, 194 (6.0%) had antibodies to SARS-CoV-2 at the baseline visit (*1*). Among these, 156 (80.4%) returned for the follow-up visit around 60 days later (range = 50–91 days). Among these 156 participants with a positive baseline serology and follow-up antibody testing performed, median age was 38 years (interquartile range [IQR] = 30–48 years), 94 (60.3%) were female, and 108 (69.2%) reported one or more symptoms of an acute infection consistent with COVID-19 between February 1, 2020 and the baseline visit. Among the 108 participants who reported symptoms, the median interval between symptom onset and baseline serology testing was 30 days (IQR = 19–40 days). Participants who reported symptoms of an acute viral illness since February had higher baseline signal-to-threshold ratios (median = 3.6; IQR = 3.1–3.9) than did those who did not report symptoms (median = 2.5; IQR = 1.5 to 3.6) (p<0.001). Among these 156 participants, 72 (46.2%) reported past RT-PCR testing for SARS-CoV-2, 46 (63.9%) of whom had positive test results; no hospitalizations were reported.

Among the 156 participants who returned for follow-up, the signal-to-threshold value for 146 (93.6%) had declined since the baseline visit, including 44 (28.2%) participants who experienced seroreversion (Table 1) (Supplementary Figure, https://stacks.cdc.gov/view/cdc/97358), with antibody levels falling below the threshold for positivity. Among 108 participants who reported previous COVID-19–compatible signs or symptoms, 21 (19.4%) seroreverted, compared with 23 (47.9%) of 48 of participants who did not report symptoms (p<0.001). Among 72 participants with previous RT-PCR testing, one (2.2%) of 46 with a positive test result versus seven (26.9%) of 26 with a negative test result seroreverted. Seroreversion occurred in 64.9% (37 of 57) of participants with a low antibody response (baseline signal-to-threshold value = 1.0–2.9) and 7.1% (seven of 99) of participants with a high antibody response (baseline signal-to-threshold value ≥3.0) (p<0.001) (Figure). A higher baseline signal-to-threshold ratio was associated with lower odds of seroreversion at the follow-up visit (adjusted odds ratio [aOR] for a 1-unit increase in signal-to-threshold ratio = 0.29; 95% CI = 0.18–0.46) (Table 2). In this model, a 10-year increase in participant age was associated with higher odds of seroreversion (aOR = 1.74; 95% CI = 1.06–2.85). Compared with non-Hispanic White participants, odds of seroreversion were lower among non-Hispanic Black participants (aOR = 0.11; 95% CI = 0.15–0.76) and Hispanic participants (aOR = 0.10; 95% CI = 0.01–0.88).

**TABLE 1:** Antibody signal-to-threshold ratio of panimmunoglobulin reactivity to SARS-CoV-2 full length S protein enzyme-linked immunosorbent assay among frontline health care personnel from a baseline visit (April–June 2020) to a follow-up visit approximately 60 days later,* overall and by baseline antibody level (N = 156) — 13 academic medical centers,^†^ United States, April–August, 2020

Baseline signal-to-threshold ratio	No.	Baseline signal-to-threshold ratio, median (IQR)	Follow-up signal-to-threshold ratio, median (IQR)	No. (%) who seroreverted
**All**	**156**	**3.4 (2.3–3.8)**	**2.6 (0.9–3.2)**	**44 (28.2)**
**Low positive (1.0–2.9)**	57	1.6 (1.3–2.4)	0.8 (0.5–1.2)	37 (64.9)
**High positive (≥3)**	99	3.7 (3.5–5.3)	3.1 (2.6–3.3)	7 (7.1)

**Abbreviation:** IQR = interquartile range.

* Range = 50–91 days. The population included 156 frontline health care personnel in the United States from 13 academic medical centers in 12 states who tested positive for SARS-CoV-2 antibodies (signal-to-threshold >1.0) at the baseline visit and underwent repeat testing at the follow-up visit.

**^†^** Harborview Medical Center (Washington), Oregon Health & Science University (Oregon), University of California Los Angeles (California), Hennepin County Medical Center (Minnesota), Vanderbilt University Medical Center (Tennessee), Ohio State University (Ohio), Wake Forest University (North Carolina), Montefiore Medical Center (New York), Beth Israel Deaconess Medical Center (Massachusetts), Baystate Medical Center (Massachusetts), Intermountain Medical Center (Utah), UCHealth University of Colorado Hospital (Colorado), and Johns Hopkins Hospital (Maryland).

**FIGURE:**
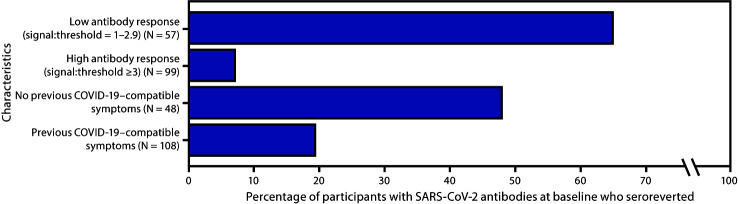
Percentage of 156 participants with SARS-COV-2 antibodies at baseline who seroreverted approximately 60 days later, by baseline antibody response* and history of COVID-19–compatible symptoms before baseline testing^†^ — 13 academic medical centers, United States, 2020 **Abbreviations:** COVID-19 = coronavirus disease 2019; ELISA = enzyme-linked immunosorbent assay. * Antibody response was categorized as high or low based on signal-to-threshold ratio of panimmunoglobulin reactivity to SARS-CoV-2 full length S protein ELISA at baseline visit ^†^ Signs and symptoms included one or more of the following reported between February 1, 2020, and the date of baseline study visit: fever (temperature >99.5°F [37.5°C]), cough, shortness of breath, myalgias, sore throat, vomiting, diarrhea, change in or loss of taste or smell, and chest tightness.

**TABLE 2:** Baseline characteristics associated with SARS-CoV-2 seroreversion (multivariable logistic regression model)* among frontline health care personnel^†^ (N = 156) — 13 academic medical centers, United States, 2020^§^

Characteristic	Odds ratio (95% CI)	
	Unadjusted	Adjusted^¶^
Signal-to-threshold ratio at baseline visit, 1-unit change**	0.34 (0.23–0.51)	0.29 (0.18–0.46)
**Age, 10-yr change††**	1.13 (0.83–1.55)	1.74 (1.06–2.85)
**Time from baseline visit to follow-up visit antibody testing, 1-wk change^§§^**	1.66 (1.24–2.21)	2.23 (1.46–3.40)
**Female sex**	0.93 (0.46–1.90)	1.49 (0.49–4.50)
**Race/Ethnicity**		
White, non-Hispanic	Referent	Referent
Black, non-Hispanic	0.24 (0.07–0.88)	0.11 (0.15–0.76)
Hispanic or Latino	0.21 (0.05–0.98)	0.10 (0.01–0.88)
Other	0.42 (0.13–1.38)	0.37 (0.08–1.59)
**≥1 baseline medical condition^¶¶^**	1.44 (0.61–3.40)	2.70 (0.74–9.94)

**Abbreviation:** CI = confidence interval.

* A seropositive result (signal-to-threshold >1.0) at the baseline visit in the spring of 2020 and a seronegative result (signal-to-threshold <1.0) at the follow-up visit approximately 60 days later.

^†^ Persons who tested positive for SARS-CoV-2 antibodies (signal-to-threshold >1.0) at the baseline visit and underwent repeat testing at the follow-up visit.

^§^ Harborview Medical Center (Washington), Oregon Health & Science University (Oregon), University of California Los Angeles (California), Hennepin County Medical Center (Minnesota), Vanderbilt University Medical Center (Tennessee), Ohio State University (Ohio), Wake Forest University (North Carolina), Montefiore Medical Center (New York), Beth Israel Deaconess Medical Center (Massachusetts), Baystate Medical Center (Massachusetts), Intermountain Medical Center (Utah), UCHealth University of Colorado Hospital (Colorado), and Johns Hopkins Hospital (Maryland).

^¶^ All variates in table were included in multivariable logistic regression model.

** This measured the odds ratio for seroreversion associated with a 1-unit difference in signal-to-threshold ratio value (e.g., 5 versus 4), comparing the higher ratio to the lower ratio.

**^††^** This measured the odds ratio for seroreversion associated with a 10-year difference in age (e.g., 60 years versus 50 years), comparing the higher age to the lower range.

^§§^ This measured the odds ratio for seroreversion associated with a 1-week difference in time to follow-up (e.g., 9 weeks versus 8 weeks), comparing the later follow-up time to the earlier follow-up time.

^¶¶^ Medical conditions included one or more of the following: asthma, chronic obstructive pulmonary disease, other chronic lung condition, chronic heart failure, coronary artery disease, diabetes mellitus, hypertension, chronic renal disease (dialysis), autoimmune disease, active cancer (not in remission), immunosuppression (undergoing active chemotherapy or taking a medication to suppress the immune system).

## Discussion

In this study of 156 frontline U.S. health care personnel who received positive SARS-CoV-2 antibody test results in spring 2020 and returned for follow-up testing approximately 60 days later, 146 (93.6%) had a decline in antibody levels between baseline and follow-up, and 44 (28.2%) had complete seroreversion, i.e., a decline of antibody to levels below the threshold for positivity. A higher percentage of those with low baseline antibody levels seroreverted (64.9%) than did those with high baseline titers (7.1%). These results suggest that a substantial proportion of persons infected with SARS-CoV-2 might have negative serologic test results in the months following infection. This has several important implications. Cross-sectional seroprevalence studies that estimate the number of persons who have been infected with SARS-CoV-2 will likely underestimate incidence because a proportion of previously infected persons will likely serorevert and thus not be counted as having been previously infected. In addition, these results challenge the notion of using serologic testing results at an individual level to designate previous SARS-CoV-2 infection. COVID-19 convalescent plasma is widely being used as a treatment for COVID-19, including through a Food and Drug Administration Emergency Use Authorization in the United States (*10*); these results demonstrate that the optimal window for collecting convalescent plasma with high levels of SARS-CoV-2 antibodies from donors who have recovered from COVID-19 might be short because of substantial decline in antibody levels within 60 days. Whether decline in SARS-CoV-2 antibodies increases risk for reinfection and disease in humans remains unknown. Humoral immunity to primary infections from a novel virus might not be as durable or strong as that to secondary infections, but memory B-cell and T-cell responses might reduce the severity of illness with repeat exposure or infection.

The findings in this report are subject to at least four limitations. First, the timing of the baseline serologic test relative to symptom onset was not standardized, which might affect baseline signal-to-threshold ratios, particularly for recently acquired infection in which antibody levels might still have been increasing. Second, the study population was derived from a convenience sample, which might result in nonrepresentativeness. Third, 38 (20%) participants were lost to follow-up, limiting size of the study population. Finally, misclassification of antibody status was possible; however, this was considered to be unlikely because of the high sensitivity and specificity of the ELISA.

In this study of frontline health care personnel at 13 medical centers who received positive SARS-CoV-2 antibody test results in spring 2020, more than one quarter were seronegative approximately 60 days after testing. Because SARS-CoV-2 antibody levels might decline in a proportion of persons following primary infection, a negative serology test does not reliably exclude previous infection. These antibody declines might not equate to loss of protective immunity or increased risk for reinfection; this was not assessed in this study. Cross-sectional seroprevalence studies to evaluate population immunity are likely to underestimate rates of previous infection because antibodies appear to only be detectable for a discrete period of time following infection.

**Summary**

**What is already known about this topic?**

Most persons develop virus-specific antibodies to SARS-CoV-2 after infection; however, the timeline of antibody decline over time is uncertain.

**What is added by this report?**

Among 156 frontline health care personnel who had positive SARS-CoV-2 antibody test results in spring 2020, 94% experienced a decline at repeat testing approximately 60 days later, and 28% seroreverted to below the threshold of positivity. Participants with higher initial antibody responses were more likely to have antibodies detected at the follow-up test than were those who had a lower initial antibody response.

**What are the implications for public health practice?**

SARS-CoV-2 antibodies decline over weeks following acute infection. Negative SARS-CoV-2 serologic results do not exclude previous infection, which has significant impacts on how serologic studies are interpreted.

## Footnotes

* Wesley H. Self and Mark W. Tenforde contributed equally to this report; Manish M. Patel and Natalie J. Thornburg contributed equally to this report.

† Participating academic medical centers and their locations were Harborview Medical Center (Washington), Oregon Health & Science University (Oregon), University of California Los Angeles (California), Hennepin County Medical Center (Minnesota), Vanderbilt University Medical Center (Tennessee), Ohio State University (Ohio), Wake Forest University (North Carolina), Montefiore Medical Center (New York), Beth Israel Deaconess Medical Center (Massachusetts), Baystate Medical Center (Massachusetts), Intermountain Medical Center (Utah), UCHealth University of Colorado Hospital (Colorado), and Johns Hopkins Hospital (Maryland).

§ Previous signs and symptoms included one or more of the following: fever (temperature >99.5°F [37.5°C]), cough, shortness of breath, myalgias, sore throat, vomiting, diarrhea, change in or loss of taste, change in or loss of smell, chest tightness.

¶ 45 C.F.R. part 46, 21 C.F.R. part 56; 42 U.S.C. Sect. 241(d); 5 U.S.C. Sect. 552a; 44 U.S.C. Sect. 501 et seq.
